# HPN Standard of Care and Long-Term Outcomes of CIF Pediatric Patients: Twenty-Eight Years' Experience in a Reference Center

**DOI:** 10.3389/fnut.2022.868273

**Published:** 2022-06-09

**Authors:** Antonella Lezo, Chiara D'Eusebio, Lorenzo Riboldi, Letizia Baldini, Marco Spada

**Affiliations:** ^1^Dietetic and Clinical Nutrition Unit, Paediatric Hospital Regina Margherita, University of Turi, Turin, Italy; ^2^Postgraduate School of Paediatrics, University of Turin, Turin, Italy

**Keywords:** chronic intestinal failure, home parenteral nutrition (HPN), standard of care, clinical outcomes, quality of life

## Abstract

**Background and Aims:**

Chronic intestinal failure (CIF) therapy changed significantly in recent decades, and both survival and complication rates improved over time. International guidelines claim that early referral of long-term home parenteral nutrition (HPN) patients to an expert center with specific standards of care may positively affect long-term outcomes. Herein, we retrospectively analyse the long-term outcomes of a cohort of pediatric patients with CIF followed-up since our Pediatric Intestinal Failure Unit foundation, in 1989.

**Methods:**

Data of the 120 children followed up at Pediatric Intestinal Failure Unit during the last 28 years were retrospectively collected. Patients' and HPN characteristics, as well as dependence, survival, and complication rates, were described.

**Results:**

Incidence and prevalence of CIF increased during the study period particularly due to the increase of HPN for non-digestive disease (NDD) CIF (47.5% of the study sample). Catheter-related bloodstream infection (CRBSI) rate decreased over the study period: 0.33 episodes/1,000 catheters days before 2011 and 0.19 episodes/1,000 catheters days afterwards. Only 1 patient out of 12 died because of HPN complications. The survival rate of patients with PDD was 98.4% at 1 year from the beginning of HPN, 96.5% at 2 years, and 93.8% from the fifth year onwards. Concerning the dependence rate, 70.6% of patients were still on HPN 1 year after the start of HPN, 63.7% at 2 years, 52.4% at 5 years, and 40.8% from the 9th year onwards, with no significant difference according to the underlying intestinal pathology. The survival rate of NDD patients was 91.2% at 1 year from the beginning of HPN, 87.4% at 2 years, and 81.9% from the third year onwards. For what concerns the enteral autonomy, it was regained by 56.7% 1 year after the start of HPN, 74.5% at 2 years, and 95.0% in the 5th year.

**Conclusions:**

Our data confirmed the importance of appropriate standards of care and suggest that applying a specific set of standards and protocols may further improve patients' outcomes and survival. Indeed, both primary and non-digestive diseases HPN showed good outcomes.

## Introduction

Intestinal failure (IF) is the reduction of gut function below the minimum required for the absorption of macronutrients and/or water and electrolytes, to the extent of mandating an intravenous supplementation to maintain health and/or growth.

The etiologies of chronic intestinal failure (CIF) include a wide range of conditions, mainly intrinsic to the gastrointestinal tract (primary digestive diseases, PDD) such as motility disorders, mucosal defects (congenital or acquired), and short bowel syndrome (SBS), or related to systemic and/or non-digestive diseases (NDD) (1, 2).

Several types of IF, requiring lifelong parenteral nutrition (PN) support for survival, may be more accurately defined as CIF or type III IF, according to the IF functional classifications (3). Type III IF is a chronic condition affecting metabolically stable patients and requiring intravenous support over months or years. It can be reversible or irreversible. In such cases, HPN is a lifesaving therapy and the best alternative to prolonged hospitalization, improving the quality of life, especially in the pediatric patients (4, 5). Previous surveys on this condition found a prevalence of 2–6.8 per 1,000,000 inhabitants in developed countries (6). In 2005, Pironi et al. reported 57 children with HPN, indicating a point prevalence of 0.7 cases/million inhabitants <18 years, in Italy (7). In 2016, another Italian study reported a prevalence and incidence of 14.12 and 1.41 cases of pediatric CIF on HPN/million inhabitants <19 years, respectively (8). Evidently, the prevalence in our country has dramatically increased over time. A comparable trend has been observed in the United Kingdom, where the prevalence of CIF has risen from 4.4/million in 1993 to 13.9/million in 2010 and 16.6/million at risk in 2012 (9).

Since the first documented use of PN in a pediatric patient in 1968, the landscape of CIF management has significantly changed (10).

Indeed, despite the wide range of underlying pathologies and the need for complex medical approaches, pediatric CIF management has evolved in recent decades, leading to improved survival rates thanks to multidisciplinary intestinal rehabilitation programs and hepato-protective strategies (11). Nationally certified expert centers for IF managing are absent in Italy, thus each region carries out artificial nutrition autonomously. Piedmont was the first Italian region to promulgate a law on HPN in 1985, strictly defining indications and standards of care. Subsequently, our center was founded in 1989 and currently boasts a 32-years experience in treating pediatric IF.

Applying a specific set of standards and protocols to HPN management may further benefit the health of CIF patients. To achieve this, the safety and efficacy of HPN services should be evaluated according to objective and measurable indicators, shared by both expert professionals and patients (12). In addition, a set of predictable clinical outcomes for patients on HPN must be designed to evaluate the efficacy of this therapy. Herein, we retrospectively analyse the long-term outcomes of pediatric patients with CIF, from the foundation of our Pediatric Clinical Nutrition Reference Center at Regina Margherita Pediatric Hospital in Turin, Italy, to the present day, to describe complications, survival rate, as well as HPN dependence rate and transition to adult care.

## Methods and Patients' Management

### Methods

Data of 120 children followed at the Intestinal Failure Unit of the Pediatric University Hospital “Regina Margherita” in Turin between 1993 and 2021 have been retrospectively collected and analyzed. We lacked data on the very first patient because the dataset started in 1993.

Collected data include sex, age, IF etiologies, characteristics and duration of HPN, number of infusions per week, incidence of complications, outcomes, school and sports attendance, occupational rate of caregivers, and transition to adult care features.

Patients were assigned to two groups based on the etiology of intestinal failure. PDD included: SBS, motility disorders, extensive parenchymal disease (including congenital diarrhoeas and active inflammatory bowel disease with a low response to biological drugs). Non-digestive disease (NDD) leading to CIF comprised: cystic fibrosis, malignant cancer, congenital cardiopathy, neurodisability, and other organ failures.

We described a well-standard of care for HPN and modalities of management of intestinal failure patients in the Piedmont region.

### Statistics

Continuous variables are reported as median value and interquartile range and compared through Student's *t*-test; categorical variables are indicated as rates and compared with Chi-square.

For each group, HPN dependence and survival rates were studied with Kaplan–Meier curves; different sub-groups curves were compared by Cox proportional-hazards model.

Statistical analysis was performed with RStudio statistical software (version 3.6.1) and Microsoft Office (version 16.54). The statistical significance was set as *p* < 0.05.

### Patients' Management

In Piedmont, diagnosis of IF and provision of HPN are regulated by a regional law that has been active since 1985. Our pediatric HPN center was founded in 1989.

All the pediatric hospitals in the region are encouraged to early referring of pediatric patients suffering from any grade of intestinal failure to the pediatric HPN center certified for long-term management of IF and provision of HPN. Appropriate PN support is then evaluated by our multidisciplinary team, composed of specialist physicians (clinical nutritionists, neonatologists, gastroenterologists, pediatric surgeons, and radiologists) as well as pharmacists, nurses, dieticians, and psychologists. Children may be discharged home once metabolically and hemodynamically stable, in adequate conditions, provided that parents are motivated to perform PN at home and the domestic environment is appropriate. The Regional Health System covers feeds, consumables, and equipment delivery at home, as well as the training of caregivers and the nursing support by a certified home care company (HCC). The parents are trained directly at home by specialized nurses of the certified HCC that is responsible for compounding and delivering HPN. The training lasts until parents feel confident with all the procedures, and when the nurses have evaluated their accuracy. Nursing support is available 24/7.

All PN solutions are infused through a central venous catheter (CVC). CVCs were all placed in our center, and the catheter type was chosen according to the expected duration of the HPN regimen and patients' characteristics. At home, the CVC is handled with a solution 2% chlorhexidine in 70% isopropyl alcohol. Since 2011, Taurolidine-citrate (Taurolock^®^) has been used in patients with a high risk of catheter-related bloodstream infection (CRBSI). TaurolockHep® has been used in patients with Port-a-Cath or with a high thrombotic risk (13).

The choice of PN bag composition is primarily based on the estimated energy requirement of each child and/or on specific needs based on the pathology. Resting energy requirements are estimated with the Schofield equation (14), while the total energy needs are obtained by multiplying the basal energy requirements by a coefficient ranging from 1.1 to 2.0, based on: physical activity level, acute and/or chronic pathology, nutritional status, growth rate and energy provided by (through) other routes (oral/enteral) (15).

Customized bags are preferred over standard ones as they are considered the standard of care. The last ones are used to facilitate hospital discharge or when PN is intended as integrative support and/or when HPN duration is estimated to be short (16).

Different types of lipids were prescribed: olive oil-based intravenous lipid emulsion (ILE) (Clinoleic^®^), composite ILE with fish oil (SMOF lipid^®^), and Omegaven^®^. The selection of the ILE was based on the estimated duration of HPN therapy, the presence or risk of liver disease, but also depending on the year of the introduction on the market of the product.

Monthly follow-up visits were performed in our outpatient clinic for the first 6 months, and every 3 months or as needed later on. At every follow-up visit, each patient undergoes a medical assessment, nutritional counseling, measurement of weight and height—which are plotted to adequate growth charts—routine blood tests and urine chemistry; oligo-elements and vitamins are dosed every 6 months, or when necessary.

Abdomen ultrasound, doppler-ultrasound of supra-aortic vessels, ultrasound bone density, or DEXA are performed annually; chest X-ray as needed.

### Complications

*Catheter-related bloodstream infection (CRBSI)* was diagnosed by taking two paired quantitative blood cultures simultaneously from both the CVC and a peripheral vein growing for the same pathogen, and by calculating the differential time to diagnosis between blood cultures drawn from the catheter and from the peripheral vein (17) or with a positive culture of CVC's tip if it is removed. The incidence of CRBSIs is expressed as the number of CRSBIs for 1,000 days of HPN.

*Catheter-related thrombosis* was defined in the presence of ultrasonography evidence.

*Established intestinal failure-associated liver disease (IFALD)* was considered if there was alkaline phosphatase >1.5 × above the normal range, total bilirubin 3–6 g/L, and abdominal ultrasound signs of liver steatosis, biliary sludge with or without gallstones (18).

## Results

**Figure 1:**
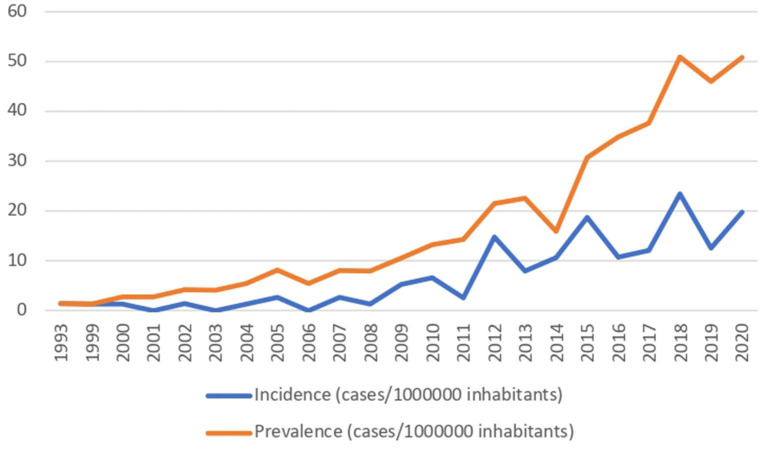
Incidence and prevalence trends of CIF along the study period.

During the follow-up period, data of 120 patients were collected; of these, 60 (50%) were female and the median age at HPN beginning was 5 years (IQR 14). Patient characteristics are shown in Table 1. Incidence and prevalence increased over the years, being 1,41 cases/1,000,000 inhabitants <19 years old in 1993, and 1,978 cases and 5,083 cases in 2020, respectively (Figure 1).

**Table 1:** Patients' characteristics.

	***n* (%)**
Number of patients	120
Female	60 (50%)
Median age at HPN beginning	5 y (IQR 14)
**Primary digestive disorders**
Number (%)	63 (52.5%)
**Etiologies**
SBS	39 (61.9%)
Extensive parenchimal disease	12 (19%)
Motility disorders	9 (14.3%)
Other primary digestive disorders	3 (4.8%)
Overall time on HPN	102,998 d (282 y)
Median time on HPN for each patient	2,5 y (IQR 5.2)
Median age at HPN start	1 year (IQR 8)
**Secondary digestive disorders**
Number (%)	57 (47.5%)
**Etiologies**
Cancers	27 (47.4%)
Cystic fibrosis	13 (22.8%)
Congenital cardiopathy	4 (7%)
Neurodisability	4 (7%)
Other pathologies	9 (15.7%)
Overall time on HPN	781 m (65 y)
Median time on HPN for each patient	6 m (IQR 15)
Median age at HPN start	14 y (IQR 12)

**Figure 2:**
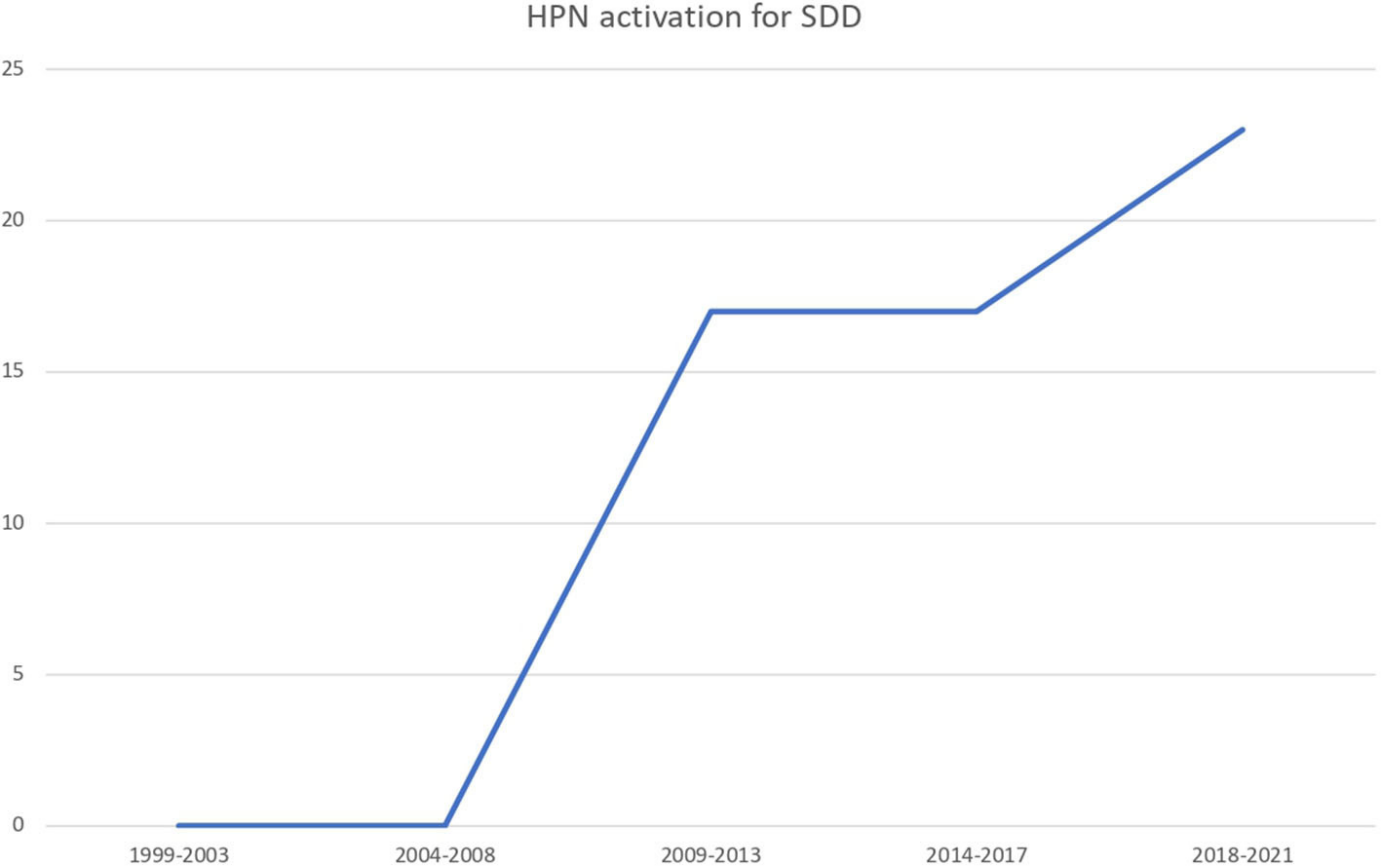
The number of HPN activation because of NPDD along the study period.

Sixty-three (52.5%) patients needed an HPN regimen because of primary digestive diseases (PDD) while 57 (47.5%) because of secondary IF (NDD). The number of HPN activation due to secondary diseases rose across the years (Figure 2). Primary etiologies included short bowel syndrome (no. 39, 61.9%), extensive parenchymal disease (no. 12, 19%), motility disorders (no. 9, 14.2%), and other PDD (no. 3, 4.8%). Secondary etiologies, or non-digestive disease leading to intestinal failure (NDD), included cancer (no. 27, 47.4%), cystic fibrosis (no. 13, 22.8%), congenital cardiopathy (no. 4, 7%), neurodisability (no. 4, 7%), and other pathologies (no. 9, 15.7%).

The median age at HPN beginning was significantly lower for patients with PDD compared to patients with secondary IF: 1-year-old (IQR 8) vs. 14-years-old (IQR 12), *p* < 0.01. Moreover, patients with PDD showed a significantly longer overall time on HPN (282 vs. 65 years, *p* < 0.01) as well as a significantly longer median time spent on HPN for each patient (−2.5 years, IQR 5.2, vs. 6 months, IQR 15. *P* < 0.01), compared to patients with NDD.

### Central Venous Catheters Characteristics

Three types of CVC were implanted in our center. The most frequent was the peripherally inserted central catheter (PICC) type, which was implanted in 62 children (51.6%), while tunneled Broviac^®^ CVC was used for 58 children (48.4%). Totally implanted Port-a-cath^®^ was used in three patients with PDD, who were teenagers at the time of CVC placement.

### Parenteral Nutrition Characteristics

The characteristics of PN infusion are shown in Table 2. The median number of PN infusions per week was 7 (IQR 1) and the median ratio between energy provided by PN and resting energy expenditure was 1.22 (IQR 0.41). The majority (59.1%) of PN bags were customized (compounded by the home care company).

**Table 2:** HPN bags characteristics.

**PN characteristics**				
Median number of PN infusion weekly	7 (IQR 1)			
Median PN calories/REE	1,22 (IQR 0.41)			
**PN bag compounding** ***n*** **(%)**				
Private company	71 (59.1%)			
Standardized bag	49 (40.9%)			
Type of lipid	SMOF n. (%)	OLIVE n. (%)	Soy-MCT	
	68 (56.6%)	51 (42.5%)	1 (0.9%)	
		SMOF n.	Other n.	*P*
Based on decade of HPN beginning	1993–2009	2 (15.4%)	12 (85.7%)	<0.05
	2010–2020	66 (62.3%)	40 (37.3%)	<0.05

The most prescribed lipid emulsion was OLIVE-based during the first period of the study (before 2011), while SMOF-lipid in the second: the difference was statistically significant (Table 2).

### Complications

**Figure 3:**
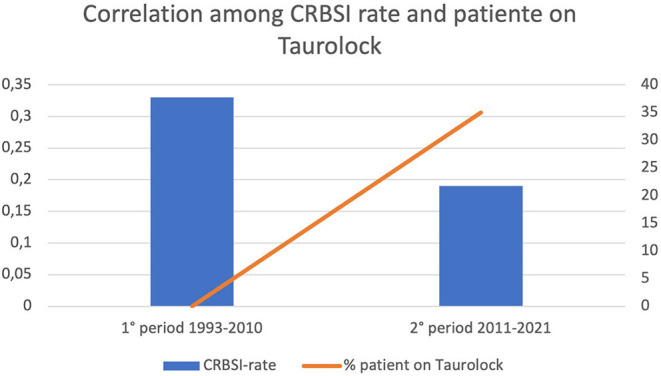
Trends of CRBSI rate reduction and percentage of patients on Taurolock.

Four patients (3.3%) developed IFALD, while 10 (8.5%) developed CVC-related thrombosis (Table 3). The overall CRBSI rate was 0.21 ep/1,000 catheter days. Over the years, the CRBSI rate descended from 0.33 episodes/1,000 catheter days during the first period of observation (1993–2010) to 0.19 episodes/1,000 catheter days during the last 10 years of observation (Figure 3). Notice that since 2011 the use of taurolidine has become clinical practice.

**Table 3:** Complications.

**Complications**	**n**°**episodes/1,000 HPN days**
**Overall CRBSI rate**	0.21
CRBSI rate - 1° decade	0.33
CRBSI rate - 2° decade	0.19
**CVC thrombosis**
Episodes	10
% of involved patients	8.5%
**IFALD**
Patients n. (%)	4 (3.3%)

### Outcomes

The overall mortality rate was 10%; among the deceased, only one patient out of twelve (who had SBS) died of HPN linked-complications.

The figure of the overall survival curves was: 94.2% 1 year after HPN start, 92.0% after 2 years, 89.7% after 3 years, and 88.1% from the fifth year onwards. Patients with SBS showed the lowest HR for death (0.07; CI 95% 0.009–0.6) though not statistically different from any of the other etiologies except for cancer (*p* < 0.05).

The dependence rate was 80.8% 1 year after starting HPN, 72.1% after 2 years, 66.6% after 3 years, 50% after 5 years, and 17.5% after 10 years. Patients with motility disorders showed an HR for weaning of 0.18 (CI 95% 0.04–0.8, *p* < 0.05).

### Outcomes in Patients With PDD

Five SBS patients underwent autologous gastrointestinal reconstruction (AGIR): three of them were treated with longitudinal intestinal lengthening and tailoring (LILT), one with isoperistaltic colon interposition, and one by serial transverse enteroplasty (STEP); the last one was subsequently weaned off HPN. None of our patients needed a referral for intestinal transplantation (ITx).

Three patients (4.8%) died; two as a result of complications linked to their underlying pathologies (severe combined immunodeficiency and Alagille syndrome, respectively) and one because of a HP-related complication (Candida-related CRBSI in a 16-month-old patient).

**Figure 4:**
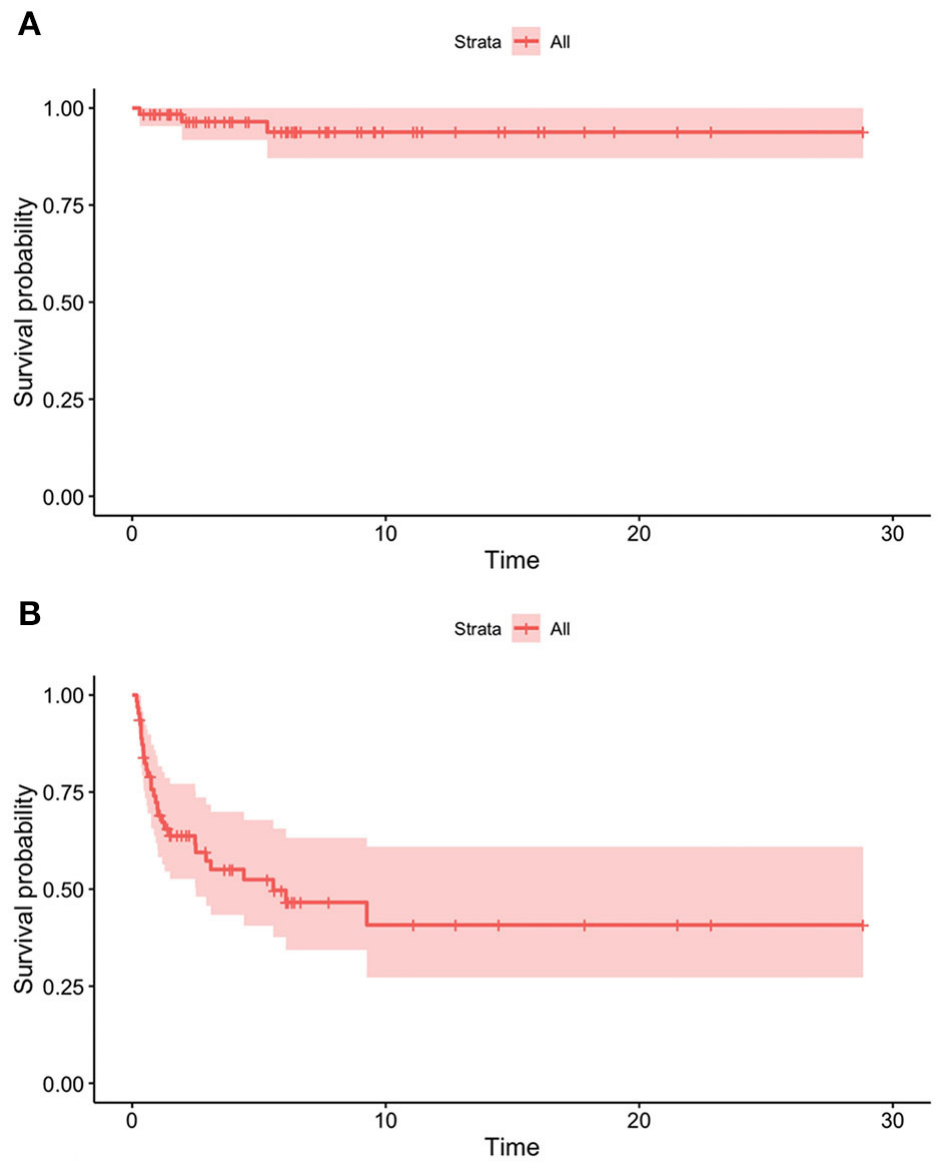
Survival **(A)** and dependence **(B)** rates of patients on HPN because of primary CIF.

The overall survival rate was 98.4% at 1 year from the HPN start, 96.5% at 2 years, and 93.8% from the fifth year onwards (Figure 4A). Dependence rates went from 70.6% in the first year after initiating HPN to 63.7% at 2 years, 52.4% at 5 years, and 40.8% from the 9th year onwards (Figure 4B). A 9-year-old patient with SBS was weaned off after 28 weeks of Teduglutide treatment (0.5 mg/kg/day) and is currently still on GLP-2 treatment.

**Figure 5:**
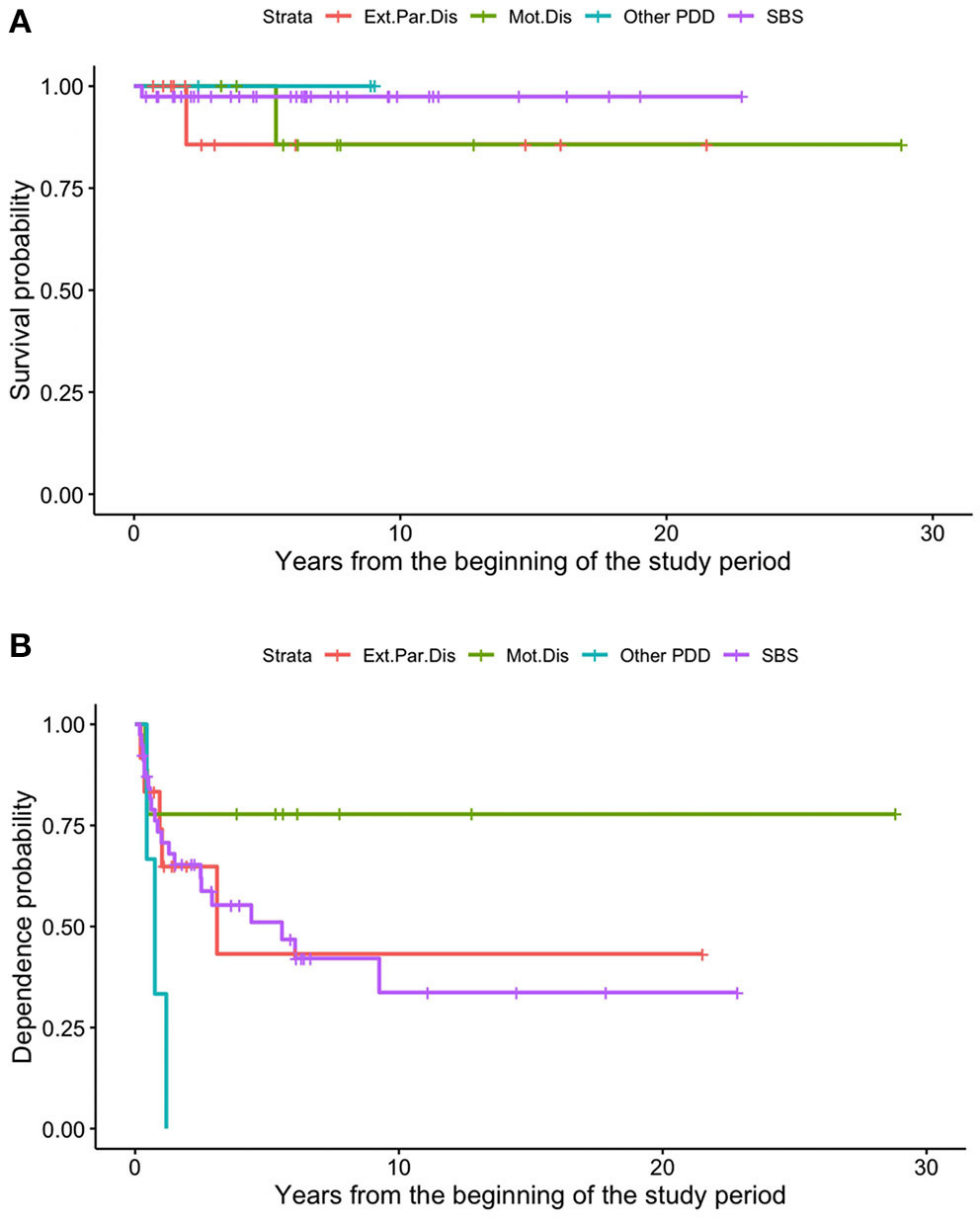
Different survival **(A)** and dependence **(B)** rates of patients on HPN because of primary CIF, based on the underlying disease.

In our sample, survival and HPN dependence did not differ significantly among patients with different intestinal pathologies (Figures 5A,B).

### Outcomes of Patients With NDD

**Figure 6:**
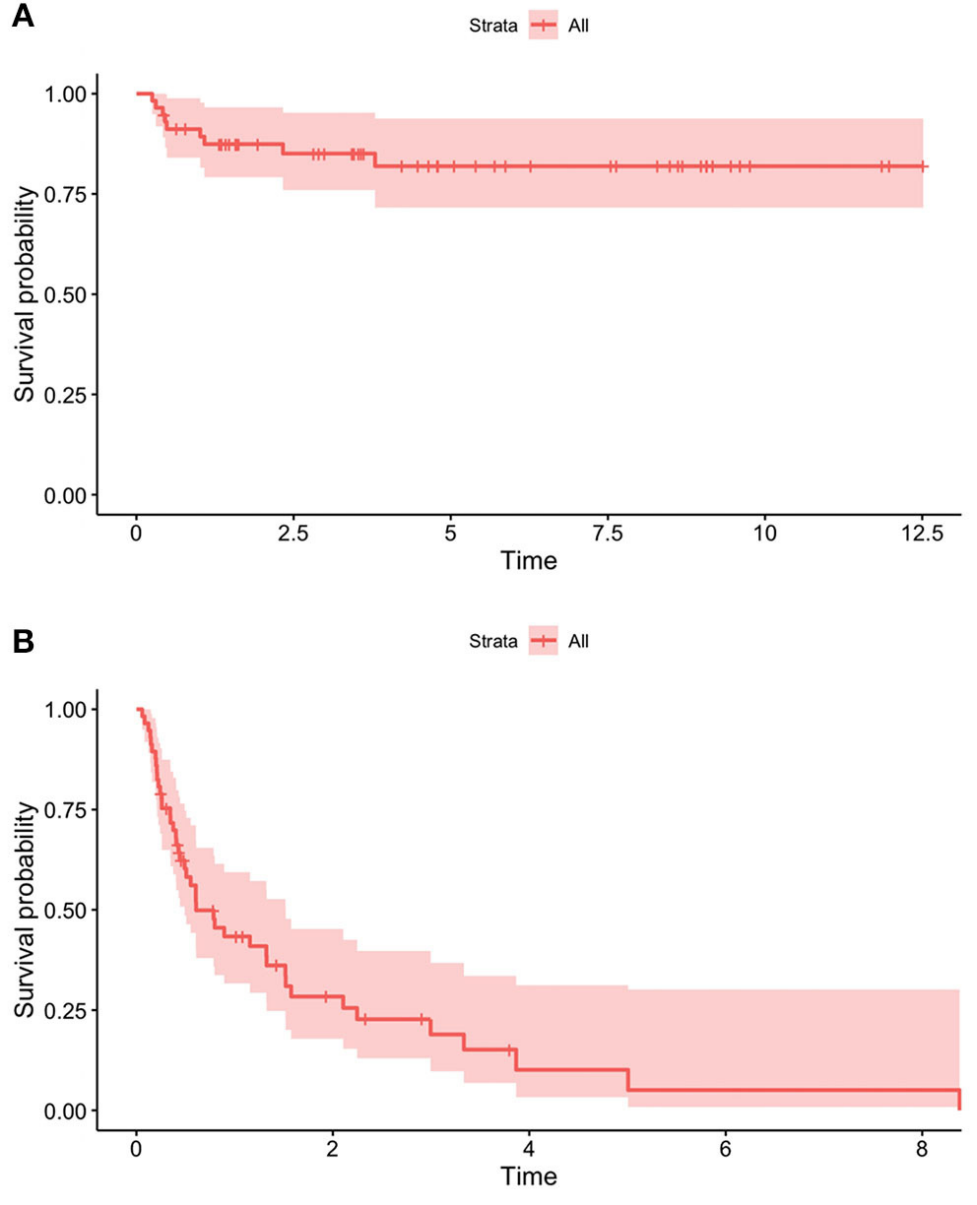
Survival **(A)** and dependence **(B)** rates of patients on HPN because of secondary CIF.

**Figure 7:**
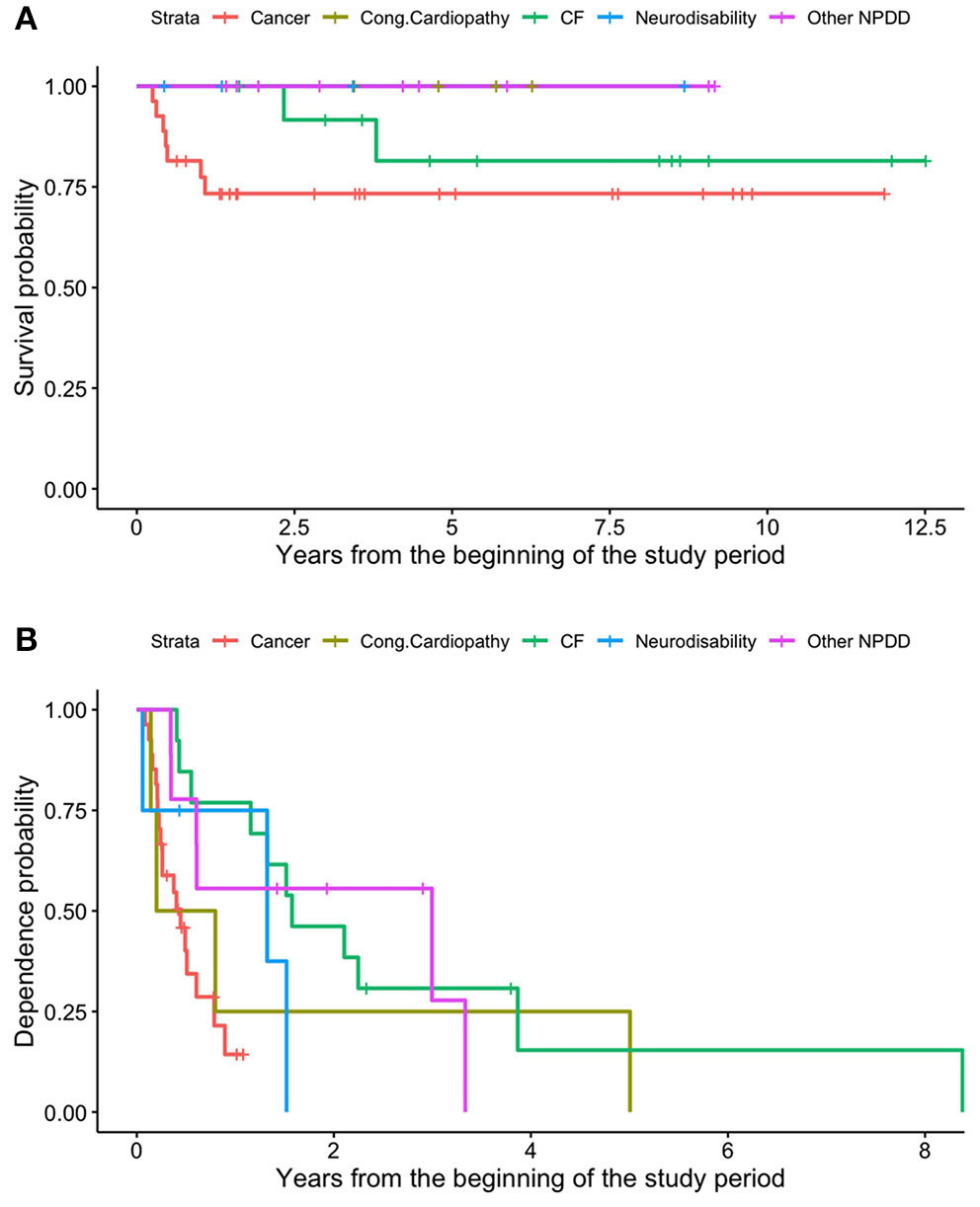
Different survival **(A)** and dependence **(B)** rates of patients on HPN because of secondary CIF, based on the underlying disease.

Nine patients (15.8%) died; of these, seven were affected by malignant cancer and two by cystic fibrosis (both patients required pulmonary transplant). The survival rate was 91.2% in the first year of HPN, 87.4% at 2 years, and 81.9% from the third year onwards (Figure 6A). As regards enteral autonomy, it was regained by 56.7% 1 year after the start of HPN, 74.5% at 2 years, and 95.0% in the 5th year. None of our patients with NDD remained on HPN for more than 8 years (Figure 6B). No statistically significant differences were detected in the probability of both dying and being weaned off HPN between the NDD (Figures 7A,B).

### Quality of Life

QoL has been indirectly evaluated through the collection of data regarding school and sports attendance, holidays, and caregivers' employment rate. Ninety percent of patients attended school; 48% of eligible patients played sports and 81% had holidays outside their region. Sixty-seven percent of the caregivers (of whom 99% were mothers) were employed.

### Transition to the Adult Care Center

Seven of our patients (four with SBS, two with motility disorders, and 1 affected by tufting enteropathy) have been transferred to the adult IF unit, a service provided by the same University Hospital. The median transition procedure duration was 6 months (IQR 4). Unfortunately, we could not provide data on post-transitional outcomes.

## Discussion

Both HPN incidence and prevalence in our region have dramatically increased over the years, from 1.41 cases/1,000,000 inhabitants in 1993 to 50.83 cases/1,000,000 inhabitants in 2020 <19 years, as shown in Figure 1. IF prevalence in our region was slightly higher than the national one: indeed, the reported Italian prevalence of CIF in 2016 was 14.12, while the incidence was 1.41 cases of pediatric CIF on HPN/million inhabitants younger than 19 years (8). On one hand, this may be related to the good practice offered by the IF center, which permits carrying on PN at home, reducing hospitalization for all the potential candidates. On the other hand, similar trends are reported in the United Kingdom, where the prevalence of CIF has risen from 4.4/million in 1993 to 13.9/million in 2010 and 16.6/million at risk in 2012. Also, Goulet et al. reported a 43.7% increase in the number of children and adolescents enrolled in HPN programs in France over the 5 years of their study period (19).

Over the years, there has been a remarkable increase in patients requiring HPN, which may be explained by several factors.

First, this augmentation was mainly ascribable to the secondary IF group. The main reason why indications of HPN in patients with NDD were extended during the last decades, is probably related to its increased safety and improved outcomes. Indeed, even if malnutrition is common in children with cancer and the need for nutritional support is quite frequent, the use of PN in oncologic pediatric patients should be judicious and led by an experienced team, as they have multiple risk factors for HPN-related complications (CRBSI, CVC thrombosis, and IFALD) (20). Moreover, it may be hypothesized that the improved survival of patients with very complex diseases may have determined an increase in the spectrum of indications for long-term parenteral nutrition support. For instance, the presence of a center for pulmonary transplantation in our region has led to a considerable number of patients with cystic fibrosis on the waiting list for pulmonary transplantation (no. 13, 22.8%) not classically reported. Conversely, we have a small number (no. 4, 3.3%) of children with neurodisability discharged on HPN, while it reached one-quarter of the patients with non-primary digestive IF in the overall data from Italy reported by Diamanti et al. (8).

Even if incidence and prevalence increase was mainly due to the NDD group, SBS remains the leading indication for HPN (32.5% of the whole sample and 62.9% among the PDD). The number of patients with IF secondary to SBS increased both in our region and in other studies. Diamanti et al. reported SBS patients as representing 56% of the overall indications, a figure higher than those previously reported by Colomb et al. (47%) and Gandullia et al. (33.3%) (21, 22). Considering the year of publication, the progressive increase in SBS percentage may be due to the improved survival of SBS patients, including those with very short residual bowel length, and the premature infants with intestinal complications requiring long-term PN. On the contrary, thanks to the advent of biological drugs, SBS secondary to Crohn's Disease (CD) has overall decreased in pediatric cohorts and this is the case even in our sample. In our study CD patients with a low response to biologicals requiring HPN in the active phase of disease were included in the extensive parenchymal disease group.

Although the prevalence of HPN has increased, indications for ITx remained low (17). None of our patients had a referral to ITx. In realities like ours, where all HPN costs are covered by the health care system, with no expenses for the families, home PN increases, thus allowing the reduction of hospital stays and better integration of children into their social and family life.

Overall, the extension of the indications for HPN may be due to its efficacy and safety. Indeed, this study showed low complications and mortality rates. So far, the majority of studies reporting outcomes of HPN in the pediatric population have been single center and retrospective, though some larger multicenter studies have also been conducted (23, 24). Individual centers have reported improvements in mortality (25), but this has yet to be systematically established. In 2019, a systematic review, meta-analysis, and meta-regression of prospective and retrospective 175 cohorts (9,318 patients) was published, in which overall mortality was 5.2% per year, mostly linked to sepsis and IFALD. Sepsis was the primary modifiable factor associated with mortality and was also predictive of IFALD and liver failure (26). That is the reason why the taurolidine lock procedure was one of the most relevant breakthroughs in the management of HPN in pediatric patients over the last few years (27, 28), with a reported decrease in CRBSI rate from 8.6 to 1.1 episodes per 1,000 days of PN (29). We observed a trend of reduction in the CRBSI rate between the first and the second decade of the study period (Figure 3), possibly due to the increase in the number of patients using taurolidine CVC locks, as reported in other recent studies (19). However, the overall infection rate in our sample was minimal (0.33 episodes/1,000 catheter days), suggesting that, even if Tauro-lock procedure is an important tool, HPN management should be based on a high-quality formal training of the caregivers as well as on the use of the most recent line connectors and tools. The very low CRBSI rate in our patients may be one of the reasons for the absence of indications for intestinal transplantation.

The other main contributor to nutritional failure and intestinal transplantation is IFALD (30). In this study, 3.3% of patients showed IFALD; of these, 3 ultrashort bowel patients developed IFALD before 2008, while one patient with congenital immunodeficiency evolved in IFALD due to numerous sepsis. Even if it is difficult to compare the IFALD incidence with other reports in the literature, due to a non-uniform choice of definition (31), our data is in line with what was reported by Olivier Goulet et al. (19). Several reasons may explain our low rate of IFALD, HPN standard of care being the most important one in our opinion: HPN and global IF multidisciplinary management by certified centers led to similar scenarios in our region and in France.

Indeed, all patients at risk of liver disease received fish oil-based lipid emulsion (Omegaven^®^) before 2010 and composite ILE with fish oil (SMOFlipid^®^) afterwards. The number of patients using this ILE was significantly higher in the second period of this study (62.3 vs. 15.4%). The remaining patients used olive oil-based ILE (Clinoleic^®^). This trend is in line with other studies that reported predominant use (>80% of patients) of SMOFlipid^®^ (19). The use of this composite ILE, which has been shown to prevent or reverse cholestasis, is a standard of care in most European centers (32).

Altogether, HPN has proved to be a safe treatment with an increased probability of survival, whose risk of death grows in the absence of a specialist team and appears to be greater during the early stages of treatment (22, 33). A study conducted in another Italian center in 2011 revealed a mortality rate of 13.9%, despite PN duration and complications (21), while another recent study reported a mortality rate of 8.1% (19). The overall mortality rate in our study was 10%, with oncologic patients accounting for seven out of 12 deaths, while the mortality rate in PDD on HPN was 4.8% (three out of 63 patients), but only 1 patient died of HPN-related complication (CVC candida Albicans infection).

Providing a high quality of life for the patients should be the main priority, and ensuring low complications rate and survival. Given that this is a retrospective analysis, we have not specifically performed inquiries on quality of life; however, data gathered about the rate of occupation of mothers (67%) and school attendance of children (90%) highlights a good administration of daily life despite HPN dependence. The vast majority of families (>80%) involved in this study were able to spend holidays outside of the region and almost half of the eligible patients are involved in a variety of sports. Although the improvement in the quality of life for patients on HPN is unquestionable, intestinal rehabilitation remains the main aim of this treatment. In our center, we observed an adequate weaning rate with no indication for intestinal transplantation; instead, AGIR helped four patients reduce PN requirements; one patient was weaned off, while two patients underwent LILT, and one with colon interposition experienced a reversal of cholestatic liver disease after surgery, in line with current literature (8).

Teduglutide, an analogous of the intestinotrophic hormone glucagon-like-peptide 2 (GLP-2) secreted by the intestinal L-cells, is becoming a possible therapeutic option for patients with SBS-related CIF (34). One of our patients was weaned off HPN, without adverse events. Data on the long-term effects of teduglutide will shine a light on whether there could be a further improvement in the dependence rate.

As far as HPN-dependent patients are concerned, the transfer to adult HPN centers remains challenging (35, 36). Seven out of 120 patients have been transferred to the adult center: in Italy, to our knowledge, our reality is the sole one that allows the transition of not only clinical reports but also PN facilities and protocols.

In conclusion, early referral of long-term PN patients to an expert center may reduce PN-associated complications and positively affect long-term outcomes, as claimed by international guidelines. Also, applying a specific set of standards and protocols to HPN management further improves outcomes and survival in both primary and secondary IF patients. The reported outcomes and the quality of life of 20 years of experience at our pediatric HPN center confirmed the importance of appropriate standards of care.

## Data Availability Statement

The raw data supporting the conclusions of this article will be made available by the authors, without undue reservation.

## Ethics Statement

Ethical review and approval was not required for the study on human participants in accordance with the local legislation and institutional requirements. Written informed consent to participate in this study was provided by the participants' legal guardian/next of kin.

## Author Contributions

AL and CD'E: conceptualization and methodology. CD'E and LR: data collection. AL, CD'E, LR, and LB: writing. AL and MS: supervision. All authors have read and agreed to the published version of the manuscript.

## Conflict of Interest

The authors declare that the research was conducted in the absence of any commercial or financial relationships that could be construed as a potential conflict of interest.

## Publisher's Note

All claims expressed in this article are solely those of the authors and do not necessarily represent those of their affiliated organizations, or those of the publisher, the editors and the reviewers. Any product that may be evaluated in this article, or claim that may be made by its manufacturer, is not guaranteed or endorsed by the publisher.
